# Children with Chronic Suppurative Lung Disease Have a Reduced Capacity to Synthesize Interferon-Gamma *In Vitro* in Response to Non-Typeable *Haemophilus influenzae*

**DOI:** 10.1371/journal.pone.0104236

**Published:** 2014-08-11

**Authors:** Susan J. Pizzutto, Stephanie T. Yerkovich, John W. Upham, Belinda J. Hales, Wayne R. Thomas, Anne B. Chang

**Affiliations:** 1 Menzies School of Health Research, Charles Darwin University, Brinkin, Northern Territory, Australia; 2 Queensland Lung Transplant Service, The Prince Charles Hospital, Chermside, Queensland, Australia; 3 School of Medicine, The University of Queensland, St Lucia, Queensland, Australia; 4 Department of Respiratory Medicine, Princess Alexandra Hospital, Wooloongabba, Queensland, Australia; 5 Telethon Kids Institute, The University of Western Australia, Crawley, Western Australia, Australia; 6 The Department of Respiratory Medicine, Royal Children's Hospital, Herston, Queensland, Australia; 7 Queensland Children's Medical Research Institute, Royal Children's Hospital, Herston, Queensland, Australia; Imperial College London, United Kingdom

## Abstract

Chronic suppurative lung disease (CSLD) is characterized by the presence of a chronic wet or productive cough and recurrent lower respiratory infections. The aim of this study was to identify features of innate, cell-mediated and humoral immunity that may increase susceptibility to respiratory infections in children with CSLD. Because non-typeable *Haemophilus influenzae* (NTHi) is commonly isolated from the airways in CSLD, we examined immune responses to this organism in 80 age-stratified children with CSLD and compared their responses with 51 healthy control children. Cytokines involved in the generation and control of inflammation (IFN-γ, IL-13, IL-5, IL-10 at 72 hours and TNFα, IL-6, IL-10 at 24 hours) were measured in peripheral blood mononuclear cells challenged *in vitro* with live NTHi. We also measured circulating IgG subclass antibodies (IgG_1_ and IgG_4_) to two *H. influenzae* outer membrane proteins, P4 and P6. The most notable finding was that PBMC from children with CSLD produced significantly less IFN-γ in response to NTHi than healthy control children whereas mitogen-induced IFN-γ production was similar in both groups. Overall there were minor differences in innate and humoral immune responses between CSLD and control children. This study demonstrates that children with chronic suppurative lung disease have an altered systemic cell-mediated immune response to NTHi *in vitro*. This deficient IFN-γ response may contribute to increased susceptibility to NTHi infections and the pathogenesis of CSLD in children.

## Introduction

Bronchiectasis and chronic suppurative lung disease (henceforth collectively termed CSLD) describe a syndrome of persistent or recurrent respiratory symptoms predominantly characterized by chronic productive or wet cough. CSLD is increasingly recognized as an important chronic respiratory disorder affecting children [1]–[4] and adults [5] globally and may represent a precursor to bronchiectasis [1].

Non-typeable *Haemophilus influenzae* (NTHi) are Gram-negative bacteria commonly associated with chronic upper and lower respiratory disease. It is the dominant species isolated from the lower airways of children and adults with chronic respiratory symptoms [6]–[8]. However, NTHi is also a commensal organism in healthy adults [9] and children [10] and as healthy adults and children both develop antibodies against NTHi [11], [12], the relationship between host and bacterium and the transition from commensal organism to pathogen is likely influenced by a complex interaction of host and bacterial factors.

One such host factor identified as important in adults is the cell-mediated immune response. Altered NTHi-specific cytokine responses, including Th2-skewed cytokine profiles have been reported in adults (>50 years of age) with established bronchiectasis or COPD and impaired lung function [11], [13]. However, it is unclear whether these alterations were involved in disease induction, or rather arose as a consequence of systemic inflammation in adults with chronic, severe disease [14], [15]. A study in children with milder disease of short duration may help elucidate some of these unresolved issues.

In the absence of published data to explain the susceptibility of some children to recurrent lower respiratory infections, we characterized systemic immune responses to NTHi in children with CSLD and healthy children. Our key outcome measures included NTHi-specific cytokine profiles (24 hour and 72 hour) *in vitro* and serum antibodies specific for the *H. influenzae* outer membrane proteins (OMP) P4 and P6. In this study we describe these profiles and identify key differences which may contribute to an increased susceptibility to lower respiratory infections in children.

## Materials and Methods

### Study population and sample collection

Eighty children (aged≤10 years) undergoing chest computed tomography (CT) scan and flexible bronchoscopy for suspected CSLD (CSLD group) and 51 age-matched children without acute infection or clinical history of respiratory or chronic illness (healthy control, HC group) were prospectively recruited (2008–2011) from the Royal Darwin Hospital (RDH), Darwin, Northern Territory (NT), Australia.

All children in the study group were clinically stable (absence of respiratory exacerbation) at the time of sample collection. Blood and bronchoalveolar lavage (BAL) for clinical and research investigations were collected at the time of intravenous access (i.e. at the start of general anesthesia), prior to chest CT scan/bronchoscopy. Clinical and socio-demographic data were collected using standardized data collection forms. Routine clinical investigations [1] were performed using the regional reference laboratory (RDH) and subsequently two children were excluded from analysis following a diagnosis of primary immunodeficiency (final n = 80). Radiographic diagnosis of bronchiectasis was made by the pediatric respiratory physician (AC). *Haemophilus influenzae*, *Streptococcus pneumoniae* and *Moraxella catarrhalis* culture and identification were performed by our laboratory (diagnostic threshold >10^4^ CFU/ml [6]). *Pseudomonas aeruginosa* and *Klebsiella pneumoniae* culture and identification were performed by the diagnostic laboratory at the Royal Darwin Hospital.

Healthy controls (absence of a history of chronic respiratory, other non-respiratory illness and no acute illness within 4 weeks) were enrolled primarily through RDH elective surgery list. They did not have the same clinical work up as the CSLD group (including chest CT, bronchoscopy) as these investigations were not clinically indicated for their procedure. Blood was collected under the same conditions as described for the group with CSLD, prior to any procedure. BAL was not collected from the control children.

The Human Research Ethics Committee (Northern Territory Department of Health and Menzies School of Health Research) approved this study (#07/63). The children were enrolled following written informed consent from the parent/carer.

### NTHi preparation

A single NTHi strain originally isolated from the sputum of an adult with pneumonia (confirmed by the Phadebact Haemophilus Test (Bactus AB, Huddinge, Sweden) and by PCR [16]) was used for this study. The immunogenicity of this strain and optimization of culture conditions were first determined in peripheral blood mononuclear cell (PBMC) cultures from healthy adults. A pilot study of 19 additional NTHi strains indicated that the strain used in this study was representative of NTHi-specific cytokine responses in a healthy adult.

NTHi was grown on chocolate agar plates overnight at 37°C, 5% CO_2_. Brain heart infusion broth supplemented with hemin (30 mg/L), nicotinamide adenine dinucleotide (30 mg/L) and glycerol (4%) was inoculated with isolated colonies and incubated with shaking at 37°C overnight. This starter culture was used to inoculate a large volume broth culture and grown to an optical density at 600 of 0.47 units. Single use aliquots were stored in 20% heat-inactivated fetal bovine serum (FBS) at −80°C. The same batch of NTHi was used in all experiments. Viability was quantified by plating serial dilutions on chocolate agar and batch viability was routinely monitored.

### Peripheral Blood Mononuclear Cell (PBMC) isolation

Venous blood was collected into preservative-free heparin and processed within 2 hours. PBMC were isolated by centrifugation over Ficoll-Paque Plus (GE Healthcare Bio-Sciences AB, Sweden) and cryopreserved in liquid nitrogen in 10%DMSO/heat-inactivated FBS. These techniques have been successfully used in studies of immune function in large human cohorts [17], [18]. Matched plasma for antibody analysis was stored at −80°C.

### PBMC challenge assays

In order to measure adaptive, cell mediated immune function, PBMC were resuspended in AIM-V serum-free medium (Gibco Life Technologies, Australia) supplemented with 2-mercaptoethanol (0.04 mM final concentration), and cultured in duplicate at 2.5×10^5^ cells per well in 96 well plates with either live NTHi (4×10^6^ CFU/ml final concentration), PHA (2 µg/ml; Sigma, Missouri, USA) or medium alone for 72 hours at 37°C, 5% CO_2_. IFNγ, IL-13, IL-5 and IL-10 were measured in supernatant collected at 72 hours. Where sufficient cells were available, innate immune responses (IL-6, TNFα and early IL-10) were also assessed. PBMC were cultured in RPMI 1640 with 10% non-heat inactivated FCS and NTHi (4×10^6^ and 4×10^5^ CFU/ml final concentration), or medium alone for 24 hours at 37°C, 5% CO_2_. Supernatants were collected and stored at 4°C for up to 7 days prior to cytokine quantification.

### Quantification of cytokines

Cytokines were measured from the 72 and 24 hour culture supernatants using dissociation-enhanced lanthanide fluorescent immunoassays (DELFIA) as described previously [19]. Quantitative standard curves were generated from serial dilutions of recombinant human cytokine proteins and included on each plate. The limit of detection for all cytokines was 10 pg/ml; samples with concentrations below the limit of detection were assigned the value of 1 pg/ml. The data is presented as delta concentrations (amount of cytokine from NTHi challenge minus nil challenge). Children were identified as responders if cytokine production was at least two-fold greater than the nil treatment or two-fold above the assay limit of detection (whichever was greater).

### Immunoglobulin levels

IgG_1_ and IgG_4_ specific for the recombinant outer-membrane proteins (OMP) P4 and P6 from *H. influenzae* were quantified from plasma by Dissociation Enhanced Lanthanide Fluorescent Immunoassay (DELFIA; PerkinElmer) as previously described [20], [21]. Negative control plasma for each OMP/antibody combination were included on each plate. Plasma IgE was measured using ELISA. Children were identified as responders if their antibody level was at least two-fold greater than the mean concentration of the negative control plasma.

### Data analysis

For the primary analysis, the children were stratified by age (<18, 18–36, 37–60 and >60 months) and the data analyzed according to respiratory diagnosis (healthy control or CSLD). As a secondary analysis, the age groups were combined and potential confounding factors of the data identified and assessed in the analysis. For the secondary analysis, only cytokines which maintained a statistical difference following correction for potential confounding factors are described.

Statistical analysis was performed using STATA 13 (StataCorp, USA) and a p-value <0.05 was considered statistically significant. Group differences (by age, diagnosis of CSLD and cultural background) were assessed by the Mann-Whitney U test and categorical data were assessed by Fisher's exact test. Cytokine and antibody data were non-parametric and are presented as median with interquartile range. Potential factors that might confound analysis of the immunologic data were identified (cultural background, clinical characteristics) and regression analysis used to investigate their effects.

## Results

### Study population

The demographic characteristics of the children are shown in table 1. All of the children in the current study came from a similar geographic region, northern Australia. High generational cultural diversity is a characteristic of the population in this region and studies to determine the influence of race on the immune response are not feasible. Despite this, we found that a high proportion of children in the CSLD group were identified by their parent as having Indigenous Australian ancestry, thus we have grouped children as either having known Indigenous Australian ancestry (described as Indigenous descent) or with no, or unknown, Indigenous Australian ancestry (non-Indigenous descent).

**Table 1:** Demographic characteristics of the population by age group.

		<18 months		18–36 months		37–60 months		>60 months	
		HC	CSLD	HC	CSLD	HC	CSLD	HC	CSLD
**n**		6	18	9	38	14	12	22	12
**Age in months**	median	8	14	26	26	51	47	81	82
	(IQR)	(5–13)	(10–15)	(24–35)	(21–29)	(46–56)	(40–54)	(66–107)	(71–106)
	p value#	0.09		0.19		0.21		0.59	
**Male**	n (%)	5 (83)	9 (50)	8 (89)	22 (58)	6 (43)	5 (42)	12 (55)	6 (50)
**Indigenous descent**	n (%)	0	13 (72)	5 (56)	36 (95)	6 (43)	12 (100)	4 (18)	12 (100)

# analysis of age between HC and CSLD at each age group.

### Children with CSLD have altered 72 hour NTHi-cytokine profiles

**Figure 1:**
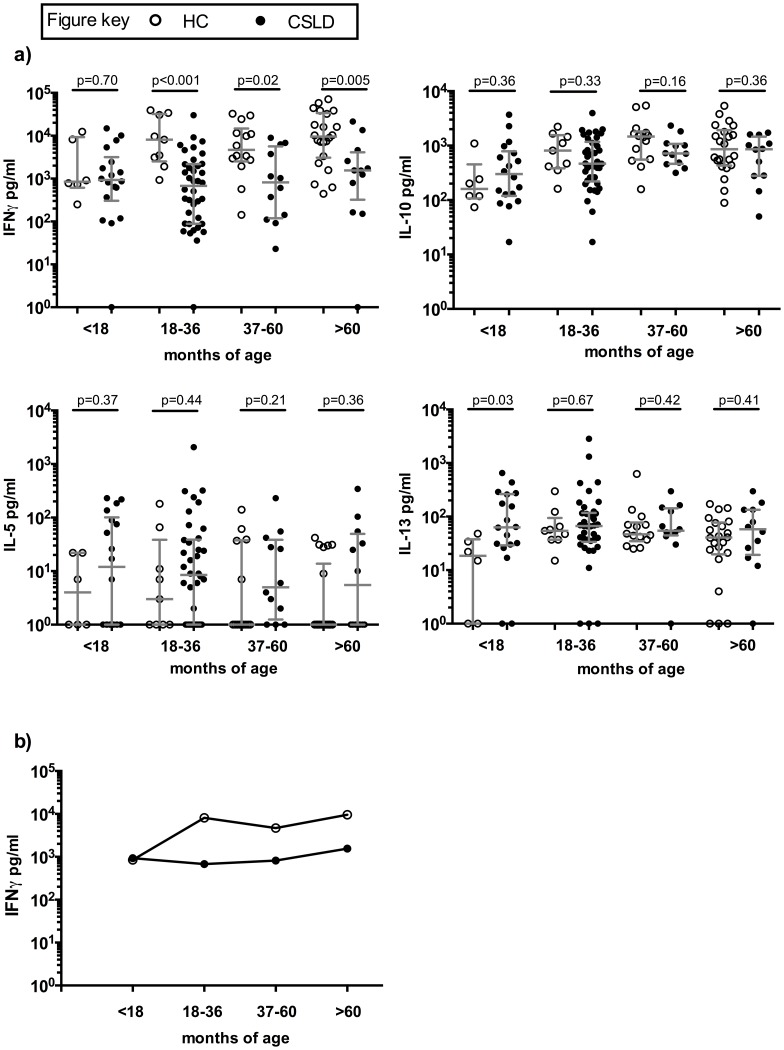
Adaptive (72 hour) cytokine production to NTHi. a) In vitro cytokine production by HC (open circles) and CSLD (filled circles) PBMC following 72 hour challenge with NTHi. Delta concentration (NTHi challenge minus nil challenge) with median and IQR. b) Trajectory of adaptive (72 hour) IFN-γ production to NTHi with age. In vitro cytokine production by HC (open circles) and CSLD (filled circles) PBMC following 72 hour challenge with NTHi. Median (delta) concentration, pg/ml.

We detected IFN-γ, IL-13 and IL-10 in the NTHi-challenged PBMC culture supernatants from almost all children (figure 1a). Whilst the number of IL-5 responders was comparatively low, the CSLD group had almost 2-fold more responders than the HC group.

For the three older age groups, PBMC from children with CSLD produced significantly less IFN-γ compared with HC children but no significant difference between groups were present in the <18 month age group (figure 1a). When all age groups were combined, IFN-γ production was also significantly lower in the CSLD group compared to HC (median (IQR) 876 (145–2536) versus 7861 (1939–17840) pg/ml, age-adjusted p<0.001; table S1).

In the HC group there was a dramatic upward trajectory in IFNγ production between the <18 month age group and the 18–36 month age groups (median 845 versus 8115 pg/ml; p = 0.06) and this level of production remained high in the older children. In contrast, IFNγ production in children with CSLD <18 months of age was similar to that seen in children with CSLD in the 18–36 month age group (median 928 versus 678 pg/ml; p = 0.36) and also similar to that seen in the older age groups (figure 1b).

Furthermore, children with CSLD aged <18 months produced higher amounts of the Th2 associated cytokine IL-13 than the age-matched HC children (median 63 versus 19 ng/ml; p = 0.03). In contrast, IL-13 production was similar in CSLD and HC children in each of the three older age groups. The production of IL-5 and IL-10 did not differ between the CSLD and HC groups across any age group.

**Figure 2:**
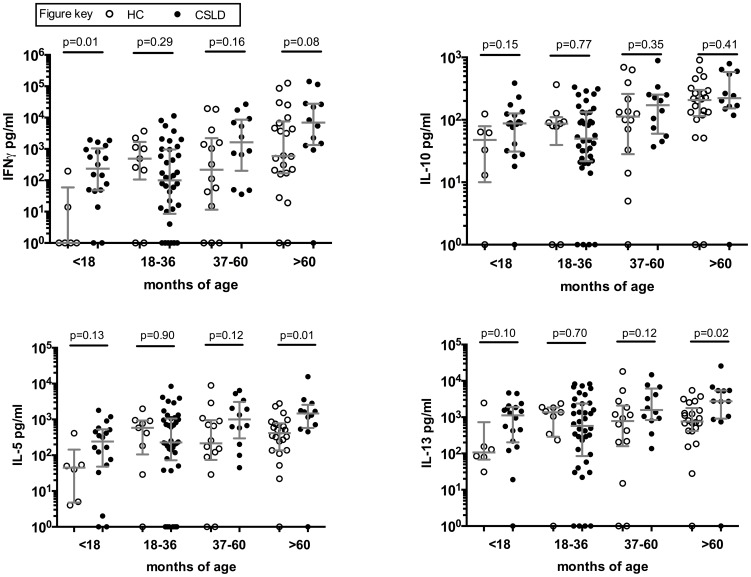
Adaptive (72 hour) cytokine production to PHA. In vitro cytokine production by HC (open symbols) and CSLD (filled symbols) PBMC following 72 hour challenge with the non-specific mitogen PHA. Delta concentration (NTHi challenge minus nil challenge) with median and IQR.

We used the non-specific mitogen PHA to test the global capacity for cytokine production and found minimal differences between CSLD and HC children (figure 2). These data indicate that the reduced capacity to synthesise IFN-γ observed in CSLD children aged >18 months is specific to NTHi and cannot be attributed to a generalised impairment in the capacity of T-cell and natural killer cells to produce these cytokines.

As CSLD was found to be prevalent in children of Indigenous descent, we investigated if this contributed to the differences in IFN-γ observed between children with CSLD and HC children. We found that Indigenous children with CSLD produced significantly less IFN-γ than age-matched Indigenous HC children (median 703 versus 3497 pg/ml; p<0.001) and non-Indigenous HC children (median 703 versus 10017 pg/ml p<0.001) (table 2). Finally, using regression analysis, Indigenous descent was not an independent predictor of IFN-γ production (table S1).

**Table 2:** Cytokine production (pg/ml; median (IQR)) from NTHi-challenged PBMC from children ≥18 months; HC Indigenous descent n = 15, HC non-Indigenous descent n = 30, CSLD Indigenous descent n = 60, CSLD non-Indigenous descent n = 2.

		Indigenous descent	non-Indigenous descent	p#
**IFN-γ (72 hr)**	**HC**	3497 (1939–33950)	10017 (3331–24437)	0.36
	**CSLD**	703 (102–2190)	557, 3858**	n/a*
	**p** ##	**<0.001**	n/a*	
**IL-13 (72 hr)**	**HC**	44 (37–123)	46 (25–66)	0.36
	**CSLD**	65 (33–129)	50, 69**	n/a*
	**p** ##	0.76	n/a*	
**IL-5 (72 hr)**	**HC**	0 (0–37)	0 (0–3)	0.07
	**CSLD**	7 (0–40)	0, 9**	n/a*
	**p** ##	0.54	n/a*	
**IL-10 (72 hr)**	**HC**	562 (352–1454)	1336 (555–2230)	**0.03**
	**CSLD**	682 (279–1109)	391, 467**	n/a*
	**p** ##	0.83	n/a*	
**TNF (24 hr)**	**HC**	656 (541–1094)	1556 (727–2086)	0.10
	**CSLD**	588 (226–1267)	181, 533**	n/a*
	**p** ##	0.53	n/a*	
**IL-6 (24 hr)**	**HC**	7154 (4221–18458)	12819 (8966–17698)	0.18
	**CSLD**	6563 (3701–10969)	2304, 2736**	n/a*
	**p** ##	0.38	n/a*	

# Analysis between Indigenous and non-Indigenous descent.

## analysis between HC and CSLD.

*p value not calculated due to small numbers.

**absolute values for the 2 participants.

### Innate (24 hour) cytokine responses to NTHi

**Figure 3:**
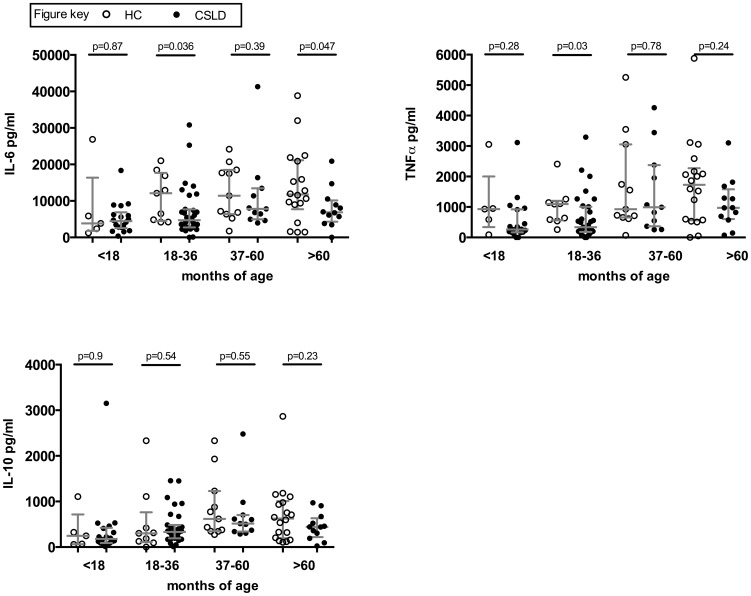
Innate (24 hour) cytokine production. In vitro cytokine production by HC (open circles) and CSLD (filled circles) PBMC following 24 hour challenge with NTHi. Delta concentration (NTHi challenge minus nil challenge) with median and IQR.

Because innate immunity may modulate adaptive immunity, we examined the strength and nature of the innate immune response to NTHi by measuring IL-6, TNFα and IL-10 production in 24 hour NTHi-stimulated PBMC (figure 3). Measurable quantities of the cytokines were produced by almost all children. Children with CSLD aged18–36 months produced less IL-6 and TNFα than HC children, but these differences were not observed in the three other age groups. IL-10 production at 24 hours was similar across all groups. When all age groups were combined, the CSLD group produced less IL-6 than the HC group (median (IQR) 5527 (3131–8733) versus11425 (4835–18458) pg/ml, age-adjusted p = 0.007; table S1).

As found with the 72 hour cytokine data, none of the 24 hour cytokine data varied in relation to Indigenous descent (table 2).

### Serum antibodies specific for H. influenzae outer membrane proteins P4 and P6

**Figure 4:**
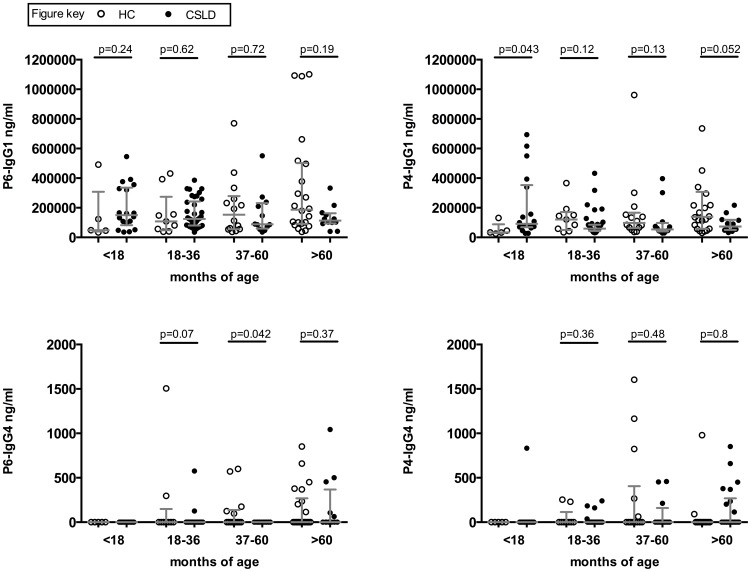
Serum IgG1 and IgG4 to outer membrane proteins P4 and P6. HC (open circles) and CSLD (filled circles). Concentration with median and inter-quartile range.

Most children had measurable serum IgG_1_ to both P4 and P6. The number producing detectable levels of IgG_4_ was low. Only minor differences were observed between the children with CSLD and the HC children (figure 4).

### Effect of patient and clinical characteristics on cytokine response

Although all children were clinically stable at the time of sample collection, 45% were receiving maintenance antibiotics for CLSD or chronic suppurative otitis media, a common co-morbidity in this population. BAL culture showed lower airway infection (growth of ≥10^4^ colony-forming units (CFU)/mL of any pathogen using standard culture methods) in 21 (25.6%) children. NTHi was the most common pathogen identified (24% of children), followed by *Streptococcus pneumoniae* (5%) and *Moraxella catarrhalis* (5%), *Pseudomonas aeruginosa* (2.5%) and *Klebsiella pneumoniae* (1%). Serum total IgE was similar between healthy controls (HC) (median 449 ng/ml; IQR 73–1142 ng/ml) and the CSLD group (641 ng/ml; 149–1623 ng/ml) (p = 0.07). All but one child were up to date with their childhood vaccination schedule. Using regression analysis, Mann-Whitney U test or Spearman's rank order correlation, we investigated whether these characteristics of the children with CSLD at the time of sample collection could explain variations in NTHi-induced cytokine production. The observed NTHi-stimulated cytokine responses were not associated with antibiotic use (including azithromycin), current isolation of NTHi or other respiratory pathogens in the BAL, evidence of parasitic infection (strongyloides), serum IgE and inflammatory cell counts in BAL and blood (table S2).

## Discussion

Despite advances in the prevention and management of pediatric respiratory infections, CSLD remains a problem in children and adults globally. This prospective, cross-sectional study is the first to compare NTHi-driven innate, cell-mediated and humoral immune responses across a large group of children with CSLD (n = 80) and healthy control children (n = 51). The most notable finding was that, in children aged ≥18 months, PBMC from those with CSLD produced significantly less IFN-γ than PBMC from their healthy peers. In contrast, in children aged <18 months, those with CSLD produced more IL-13 than the healthy control children. We found comparatively minor differences in humoral and innate immune responses. These data are consistent with our hypothesis that impaired cell mediated immune responses may contribute to the pathogenesis of CSLD in children.

Immune development begins in utero [22], [23]. Various environmental, respiratory and immune pressures drive the maturation of the Th1 response from the Th2-dominated response of neonates [24]–[26]. By 18 months of age there is a substantial rise in IFN-γ function [27] and a delay in the development of the IFN-γ response may predict Th2-associated disorders in childhood [28]. IFN-γ, primarily secreted by T lymphocytes and natural killer cells, is integral to the orchestration of antiviral and antibacterial activity and is a dominant Th1 cytokine. Furthermore impaired IFN-γ responses have been implicated in the pathogenesis of rhinovirus-induced asthma exacerbations in adults [29]. Whilst our study was not designed to investigate the mechanistic process of immune maturation, our findings have highlighted a disparity between children with CSLD and HC children in age-related IFN-γ and IL-13 production. We found a log-fold increase in IFN-γ production between HC aged <18 months compared to those >18 months. The capacity to produce IFN-γ remained high in the older age groups. These data are consistent with normal maturation of the Th1 response through early childhood. This age-related increase in IFN-γ response observed in the HC children was not reflected in the children with CSLD. Children <18 months of age with CSLD had a similar capacity for NTHi-specific IFN-γ production as their HC peers, however unlike the HC group, the CSLD group maintained a significantly lower capacity for IFN-γ production over subsequent increasing age groups. Conversely, IL-13 production was significantly higher in younger children with CSLD, with no significant difference between the two groups in older children. Whilst future longitudinal studies are required, these data support the notion that normal maturation of the cell mediated immune response is impaired in children who develop CSLD.

We also examined cytokines representative of innate immune responses in PBMC challenged for 24 hours with NTHi reasoning that altered innate immunity might provide a mechanism for altered adaptive immunity. No studies have previously addressed this issue in children with CSLD. IL-6 is an important cytokine in facilitating the transition from the innate to the adaptive response and is also important for the resolution of acute inflammation (reviewed in Jones [30]). The low IL-6 response to NTHi which we observed in children with CSLD as a group and in the 18–36 month age group is consistent with our data demonstrating an impaired cell-mediated immune response. That TNFα production was also low in 18–36 month CSLD children may point to an immaturity or impaired function of innate immune cells such as monocytes or dendritic cells. Immaturity of such antigen presenting cells would also account for low IFN-γ responses to antigenic stimulation via impaired IL-12 or IL-27 pathways [31]. The possible contribution of IL-6 and other Th1-polarising cytokines, including IL-12 and IL-27, to the pathogenesis of NTHi infections and chronic inflammation requires exploration in future longitudinal studies.

P4 and P6 are highly conserved, immunogenic outer membrane proteins ubiquitously expressed on *H. influenzae*. Given the association of OMP antibodies with protection from NTHi infection in animal models [32] and the association of low P4 and P6 IgG_1_ titres with asthma and atopy, in children [20], we investigated if children with CSLD were deficient in NTHi-specific antibodies, especially since clinical investigations found no deficiency in total IgG or any IgG subgroup overall. Consistent with published data [20], [21], most children in our study produced IgG_1_ to P4 and P6 whilst the number of children (CSLD and HC) producing IgG_4_ to either antigen was low. In contrast to published data of children with Th2-associated disorders asthma and atopy [20] we found no significant difference in P4 and P6 IgG1 levels between children with CLSD and HC children. These results plus the comparable levels of early (24 hour) and later (72 hour) IL-10 responses support the hypothesis that non-protective immunity to NTHi in children with CSLD is more closely linked to aberrant cell-mediated immune function rather than the humoral arm of the immune response.

Our finding that children with CLSD have an impaired IFN-γ response to NTHi has some interesting parallels with that described in adults with the chronic respiratory conditions associated with infection (COPD and bronchiectasis). In these studies involving smaller cohorts, King and colleagues demonstrated that persistent or recurrent lower respiratory infection with NTHi in adults with COPD (n = 39) or bronchiectasis (n = 16) was associated with high IL-13 production in the airways and low systemic IFN-γ production [13], [11]. These studies provide important data on the pathogenesis of NTHi infection in adults, however extrapolation of this data to the pediatric setting may not be appropriate. The strengths of our study, which also distinguish it from the adult studies, lie in our large sample size of young children with early onset chronic disease. 75% of our total cohort (n = 80) were aged <3.5 years and had comparatively mild disease of recent onset. The effect of age, chronic inflammation, airway remodeling and pollutants on immune responses is well documented [33]–[36]. Thus studying CSLD in children with early stage disease may be more instructive to understanding the early pathogenesis of bronchiectasis. The differences in immune function described in the current study are more likely to be involved in disease initiation rather than an effect of chronic disease and long standing tissue damage.

It is now evident that the airways are colonized with microbial flora and that respiratory illness is frequently characterized by changes in the normal microbiota [9]. Furthermore emerging evidence suggests that normal immune profiles vary between populations; children from regions of high pathogen burden have functionally distinct immune profiles compared with children from regions of low burden [23], [24]. Whilst all of the children in our study came from a similar geographic location, a large proportion of our CSLD group identified with an Indigenous Australian ancestry. Many factors beyond the scope of this study may contribute to this disparity including both biological and socio-economic determinants of health. We undertook several approaches to ensure that the differences in cytokine profiles were a result of the CSLD and not merely explained by the demographics of the population.

There are numerous host and environmental factors which may influence the immune response thus, whilst our current study has novel findings, we acknowledge some limitations. We chose to use NTHi because it is the most common bacterium in children with CSLD but it was beyond the scope of our study to examine if other pathogens also induce low IFN-γ responses. Secondly it is a cross-sectional study thus we cannot explain the cause or the effect of the observed impaired IFN-γ response in children with CSLD. Also, the association between systemic and local airway responses is unknown as limited cell numbers obtained from BAL precluded measurement of IFN-γ production by airway T-cells in our study. Nonetheless, our findings suggest that altered systemic immune responses are characteristic of CSLD, and we would argue that both local and systemic immunity are likely to be important in defending the lungs from respiratory pathogens.

In conclusion, this is the first study to compare NTHi-driven innate, cell-mediated and humoral immune responses between a large group of children with CSLD and HC children. Our key finding is that the ability to induce protective cell mediated immune responses against NTHi is compromised in children with CSLD. We found clear evidence of impaired IFN-γ responses by PBMC, *in vitro*, in children with CSLD and limited evidence of impaired innate and humoral responses. Furthermore, whilst our data on young infant responses is preliminary it suggests that this impairment begins at an early age. This study has important implications for research into management strategies for children at risk of CSLD. Effective management strategies require an understanding of host responses to respiratory pathogens and the contribution of these responses to the pathogenesis of chronic infection. Future intervention studies that can improve cell mediated immune responses along with longitudinal studies examining the relative contributions of delayed immune development, and high microbial load to the pathophysiology of CSLD in children, are required.

## Supporting Information

Table S1 a) Linear regression model of IFN-γ production at 72 hours. b) Linear regression model of IL-13 production at 72 hours. c) Linear regression model of IL-10 production at 72 hours. d) Linear regression model of IL-6 production at 24 hours.
(DOC)

Table S2 **Analysis of patient characteristics on NTHi-specific cytokine production.** # p value from analysis by Mann-Whitney U test, ## p value from analysis by ANOVA, ### p value from analysis by Spearman's rank order correlation.
(DOC)
