# Supplementary material for: Children with Chronic Suppurative Lung Disease Have a Reduced Capacity to Synthesize Interferon-Gamma In Vitro in Response to Non-Typeable Haemophilus influenzae
Source: PLoS One. 2014 Aug 11;9(8):e104236. doi: 10.1371/journal.pone.0104236 (PMC4128648; doi:10.1371/journal.pone.0104236)
Supplement: Table S1 — a) Linear regression model of IFN-γ production at 72 hours. b) Linear regression model of IL-13 production at 72 hours. c) Linear regression model of IL-10 production at 72 hours. d) Linear regression model of IL-6 production at 24 hours. (DOC) [file pone.0104236.s001.doc]

Supporting Information Table S1

a) Linear regression model of IFN-γ production at 72 hours

| **Univariate analysis** | ***β*** | ***p*** | ***95% CI for β*** |
| --- | --- | --- | --- |
| Age (months) | 0.201 | <0.001 | 0.01 – 0.03 |
| Indigenous background | -1.498 | <0.001 | -2.15 – -0.84 |
| CSLD | -1.963 | <0.001 | -2.55 – -1.38 |
| **Multivariate analysis** | ***β*** | ***p*** | ***95% CI for β*** |
| CSLD | -1.758 | <0.001 | 0.09 |
| age | 0.01 | 0.09 | -0.001 – 0.02 |

b) Linear regression model of IL-13 production at 72 hours

| **Univariate analysis** | ***β*** | ***p*** | ***95% CI for β*** |
| --- | --- | --- | --- |
| Indigenous background | 0.38 | 0.055 | -0.01 – 0.77 |
| CSLD | 0.38 | 0.046 | 0.006 – 0.76 |
| **Multivariate analysis (r^2^ =0.28)** | ***β*** | ***p*** | ***95% CI for β*** |
| CSLD | 0.25 | 0.31 | -0.23 – 0.73 |
| Indigenous background | 0.22 | 0.38 | -0.28 – 0.72 |

c) Linear regression model of IL-10 production at 72 hours

| **Univariate analysis** | ***β*** | ***p*** | ***95% CI for β*** |
| --- | --- | --- | --- |
| Age | 0.01 | 0.008 | 0.002 – 0.02 |
| Indigenous background | -0.38 | 0.07 | -0.79 – 0.03 |
| CSLD | -0.45 | 0.02 | -0.85 – -0.06 |
| **Multivariate analysis (r^2^ =0.07)** | ***β*** | ***p*** | ***95% CI for β*** |
| CSLD | -0.21 | 0.43 | -0.74 – 0.32 |
| Age | 0.01 | 0.04 | <0.001 – 0.01 |
| Indigenous background | -0.15 | 0.57 | -0.68 – 0.37 |

d) Linear regression model of IL-6 production at 24 hours

| **Univariate analysis** | ***β*** | ***p*** | ***95% CI for β*** |
| --- | --- | --- | --- |
| Age | 0.01 | 0.007 | 0.002 – 0.01 |
| CSLD | -0.50 | 0.006 | -0.85 – -0.14 |
| **Multivariate analysis (r^2^ =0.10)** | ***β*** | ***p*** | ***95% CI for β*** |
| CSLD | -0.38 | 0.04 | <0.001 – 0.01 |
| Age | 0.001 | 0.052 | <-0.001 – 0.01 |
