# Supplementary material for: Children with Chronic Suppurative Lung Disease Have a Reduced Capacity to Synthesize Interferon-Gamma In Vitro in Response to Non-Typeable Haemophilus influenzae
Source: PLoS One. 2014 Aug 11;9(8):e104236. doi: 10.1371/journal.pone.0104236 (PMC4128648; doi:10.1371/journal.pone.0104236)
Supplement: Table S2 — Analysis of patient characteristics on NTHi-specific cytokine production. # p value from analysis by Mann-Whitney U test, ## p value from analysis by ANOVA, ### p value from analysis by Spearman's rank order correlation. (DOC) [file pone.0104236.s002.doc]

Supporting Information, Table S2. Analysis of patient characteristics on NTHi-specific cytokine production. # p value from analysis by Mann-Whitney U test, ## p value from analysis by ANOVA, ### p value from analysis by Spearman’s rank order correlation

|  | **number of children (%)** | **p value** | | | |
| --- | --- | --- | --- | --- | --- |
|  |  | **IFNγ** | **IL-13** | **IL-10** | **IL-5** |
| **Antibiotic use**^#^  Azithromycin  Other | 25 (31)  11 (14) | 0.38 | 0.99 | 0.64 | 0.87 |
| **Lower airway infection**^#^  Any pathogen  non-typeable *H. influenzae* *Streptococcus pneumoniae*  *Moraxella catarrhalis*  *Pseudomonas aeruginosa*  *Klebsiella pneumoniae* | 21 (26)  16 (20)  4 (5)  4 (5)  2 (2.5)  1 (1.3) | 0.52  0.28  -  -  -  - | 0.63  0.77  -  -  -  - | 0.77  >0.9  -  -  -  - | 0.87  0.71  -  -  -  - |
| **Strongyloides serology positive** ^#^  **(n tested=72)** | 7 (9.7) | 0.64 | 0.71 | 0.70 | 0.45 |
| **Serum total IgE^##^** |  | 0.41 | 0.74 | 0.74 | 0.83 |
| **Blood inflammatory cells^###^**  **(n tested=76)**  White cell count  neutrophils  Lymphocytes |  | 0.26  0.80  0.72 | 0.84  0.47  0.53 | 0.84  0.78  0.96 | 0.85  0.73  0.39 |
| **Airway inflammatory cells^###^**  neutrophils  eosinophils |  | 0.76  0.18 | 0.90  0.88 | 0.94  0.50 | 0.80  0.33 |
